# Influence of the prosthetic arm length (palatal position) of zygomatic implants upon patient satisfaction

**DOI:** 10.4317/medoral.21033

**Published:** 2016-03-06

**Authors:** Hilario Pellicer-Chover, Juan Cervera-Ballester, David Peñarrocha-Oltra, Leticia Bagán, María Peñarrocha-Diago, Miguel Peñarrocha-Diago

**Affiliations:** 1 DDS. Professor of the Master in Oral Surgery and Implantology, Department of Stomatology, Valencia University Medical and Dental School, Valencia, Spain; 2 DDS, PhD. Associate Professor, Department of Stomatology, Valencia University Medical and Dental School, Valencia, Spain; 3 DDS. Collaborator in Oral Medicine, Department of Stomatology, Valencia University Medical and Dental School, Valencia, Spain; 4 MD, PhD, DDS. Full Professor of Oral Surgery, Department of Stomatology, Valencia University Medical and Dental School, Valencia, Spain; 5 MD, PhD. Chairman of Oral Surgery, Director of the Master in Oral Surgery and Implantology, Department of Stomatology, Valencia University Medical and Dental School, Valencia, Spain

## Abstract

**Background:**

To assess the influence of the prosthetic arm length (palatal position) of zygomatic implants upon patient comfort and stability, speech, functionality and overall satisfaction.

**Material and Methods:**

A retrospective clinical study was made of patients subjected to rehabilitation of atrophic maxilla with complete maxillary implant-supported fixed prostheses involving a minimum of two zygomatic implants (one on each side) in conjunction with premaxillary implants, and with 12 months of follow-up after implant loading. Subjects used a VAS to score general satisfaction, comfort and stability, speech and functionality, and the results were analyzed in relation to the prosthetic arm length of the zygomatic implants 12 months after prosthetic delivery.

**Results:**

Twenty-two patients participated in the study, receiving 22 prostheses anchored on 148 implants (44 were zygomatic and 94 were conventional implants). The mean right and left prosthetic arm length was 5.9±2.4 mm and 6.1±2.7 mm, respectively, with no statistically significant differences between them (*p*=0.576). The mean scores referred to comfort/retention, speech, functionality and overall satisfaction were high - no correlation being found between prosthetic arm length and patient satisfaction (*p*=0.815).

**Conclusions:**

No relationship could be identified between prosthetic arm length (palatal position) and patient satisfaction.

**Key words:**Zygomatic implants, patient satisfaction, zygomatic prosthesis, prosthetic arm length.

## Introduction

Zygomatic implants, when positioned in conjunction with premaxillary implants, can facilitate the surgical rehabilitation of patients with severe maxillary resorption (1). Furthermore, the final position of the zygomatic implant can affect the configuration of the prosthesis, because of the location and emergence of these implants at a more medial position compared to standard maxillary implants (2).

Some authors (3-6) have reported satisfactory results in patients treated with fixed dental prostheses supported by multiple zygomatic implants. However, Bothur *et al.* (7) observed mild speech deterioration among these patients. Petrovic *et al.* (8) showed that a 2 mm sagittal alteration of the maxillary incisor can cause up to 80% distortion of speech in patients wearing complete dentures, and found that speech distortion increases rapidly when the palatal plate is more than 1 mm thick. The palatal emergence of zygomatic implants placed using the intrasinusal technique requires the preparation of a prosthesis with “palatal arms” that invade the lingual space. We have found no studies in the literature on the relationship between the prosthetic arm length (palatal position) of zygomatic implants and patient satisfaction.

The aim of this study was to assess the influence of the prosthetic arm length (palatal position) of zygomatic implants upon patient comfort and stability, speech alterations, functionality, and overall satisfaction.

## Material and Methods

The present study is reported in accordance with the Strengthening the Reporting of Observational Studies in Epidemiology (STROBE) statement (9).

- Patient selection

A retrospective clinical study was made in the Oral Surgery and Implant Dentistry Division of the University of Valencia (Valencia, Spain), involving patients treated between 1998 and 2004. The study was carried out following the principles of the Declaration of Helsinki on human research. Accordingly, all patients were informed about the study and were asked to sign an informed consent document before being included. The study design was approved by the ethical review board of the University of Valencia (Ref.: H1402993407096).

Patients subjected to rehabilitation of atrophic maxillas (Cawood and Howell Class VI) with complete maxillary implant-supported fixed prostheses involving a minimum of two zygomatic implants (one on each side) in conjunction with premaxillary implants and followed-up on for 12 months after implant loading were included in the study.We excluded patients who failed to complete the questionnaire or who did not attend the follow-up examinations.

- Description of procedures

All surgeries were carried out by the same surgeon (MPD) under local anesthesia (4% articaine with 1:100,000 adrenalin [Inibsa, Lliça of Vall, Barcelona, Spain]) and sedation (1% propofol solution), with blood pressure, pulse, and oximetric monitoring by the anesthetist. Patients received zygomatic fixtures combined with conventional implants. Zygomatic implants (Nobel Biocare, Göteborg, Sweden) were placed in the malar zygoma using the procedure described by Stella and Warner (2). A further three to 6 conventional implants were inserted into the residual maxillary alveolar process. All implants remained submerged, and the second surgery was carried out three months later.

For the first week following implant placement, patients were instructed not to use provisional prostheses; thereafter they used their preexisting complete dentures as provisional prostheses during the implant healing period. The definitive screw-retained prostheses were placed four months after surgery.

- Patient-based measurements

All patients were reviewed one month after implant placement and at 6 and 12 months after delivery of the definitive prostheses. The measurements were performed by two independent observers (JCB and HPC) previously calibrated using sample instruments. The concordance study was based on calculation of the weighted kappa index.

**Figure 1:**
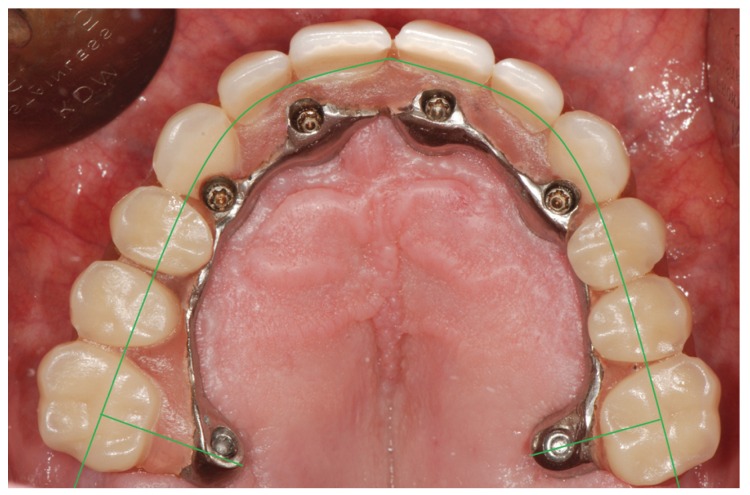
View of the reference lines and measurements used in the study of the prostheses.

We used a line on the occlusal surface of the molars and premolars and cingulum of the maxillary canines and incisors (occlusal line). Then, a perpendicular line was traced running parallel to the palatal prosthetic arm; the line was measured with precision calipers (resolution: 0.01 mm/0.0005” - precision: ± 0.1 mm) (PCE Iberica, S.L. Albacete, Spain) (Fig. 1).

To estimate patient satisfaction with the fixed prosthesis, 10-cm visual analog scales (VAS) (range 1-10) were given to each patient 12 months after prosthesis delivery. The VAS were used to score general satisfaction with the implant-retained prosthesis, comfort and stability, speech, functionality and overall satisfaction. Patients were asked to rate these aspects of their care on the VAS from 0 (totally dissatisfied) to 10 (completely satisfied). The subjects were asked to draw a vertical line at the point on the horizontal line that best represented their response (4). The patients marked the scale independently in the presence of a research assistant who offered explanations or help as needed.

## Results

Twenty-two patients (11 women and 11 men) with a mean age of 54 years (range 31-77) were included in the study. Ten patients presented class IV atrophy and 12 patients had class V atrophy according to the classification of Cawood and Howell. The opposing arch exhibited natural dentition in 5 patients and implant-supported fixed dentures in 5. Five patients had a combination of natural dentition and implant-supported fixed prostheses, and 7 patients already had an overdenture in the opposing arch. Twelve patients received four standard anterior implants, 5 received 6, one received two, three received three, and one patient received 5 implants in the residual maxillary alveolar process ( Table 1).

**Table 1:**
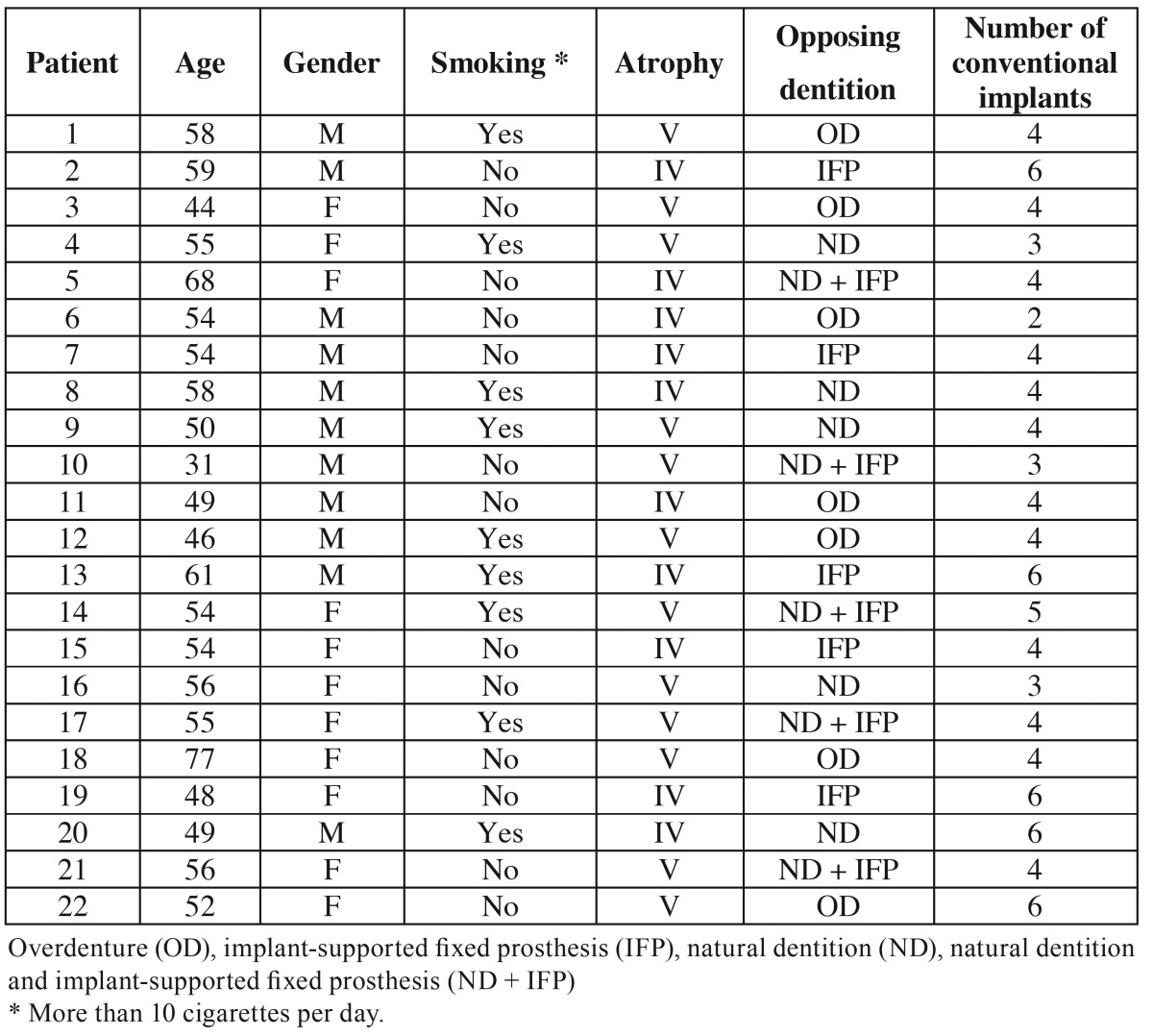
Characteristics of patients treated with zygomatic implants.

The zygomatic implant success rate was 97.7% (one implant failed during osseointegration and was replaced by a pterygoid implant) and the standard anterior implant success rate was 97.8% (two standard implants failed during the study period).

The mean length of the prosthetic arms was 6 ±2.4 mm (5.9 ±2.4 mm on the right side and 6.1 ±2.7 mm on the left). The overall satisfaction score was9.45 out of 10 (9.68 for comfort-stability, 9.36 for speech, and 9.64 for functionality).

Since the sample size was small (n=22), normality for the arm length data was checked using the Shapiro-Wilk test (*p*>0.05). Applying a t-test for paired samples, it was confirmed that the mean right side lateral length was equal to that of the left side (*p*=0.576), showing that the longest prosthetic arm and average length were very similar and led to the same results. We decided to base the data analysis on the maximum length ( Table 2).

Given that patient satisfaction generated values that were markedly asymmetrical (all patients gave scores of 7 or higher, and most gave scores of 9-10), any possible correlation to length was evaluated using Spearman’s rank correlation coefficient. The results are shown in  Table 2.

**Table 2:**
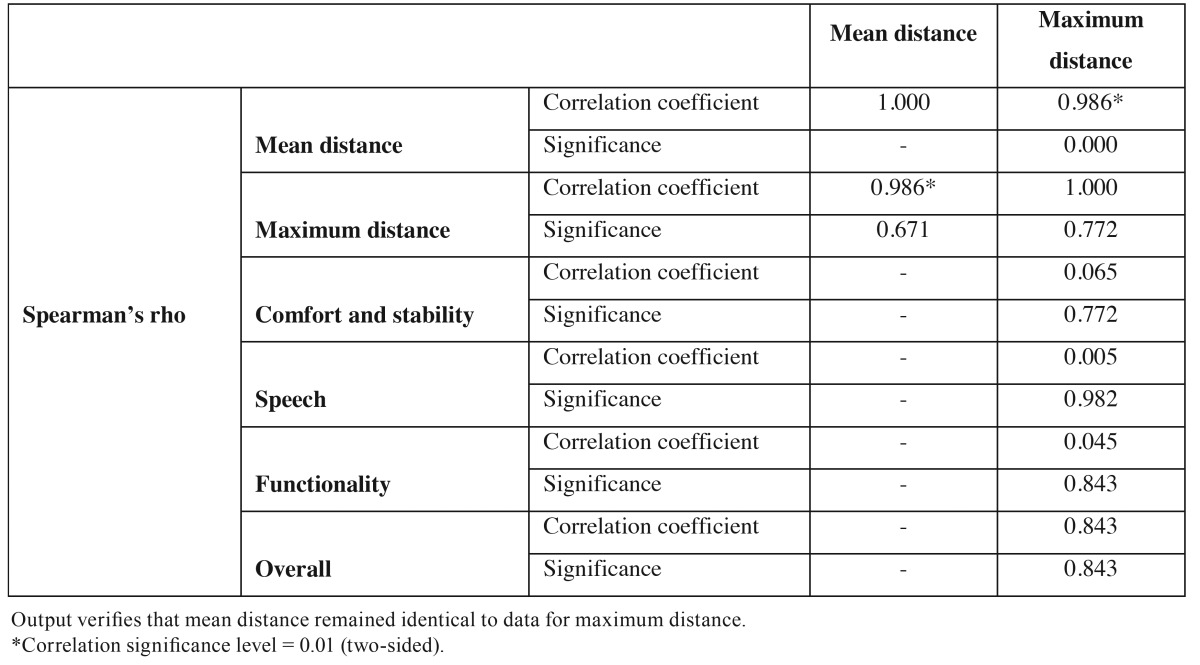
Correlations.

## Discussion

In the present study, only two of the 94 anterior implants were lost. The losses occurred during the study period. No other implant failures occurred during the follow-up period, and all prostheses remained in full function. These findings indicate a 97.7% survival rate for the zygomatic implants and a 97.8% survival rate for the anterior implants. Previously reported survival rates have ranged from 94.2-100% for zygomatic implants (1,10-12) and from 73-91.8% for anterior implants (1,13,14).

Only two studies (12,14) have evaluated the mean distance from zygomatic head center to ridge center. Aparicio *et al.* (12) obtained a distance on the right and left sides of 4.5 mm and 5.7 mm, respectively. These values were slightly lower than in the present study (5.9 ±2.4 mm on the right side and 6.1 ±2.7 mm on the left side), possible because of the reference points selected for measuring the prosthetic arms.

The present study found no significant differences in patient satisfaction; all patients gave very favorable VAS scores for the implant-supported prostheses, regardless of prosthetic arm length. These results support the findings of other authors (3,4,6,11). However, Aparicio *et al.* (12) observed that four zygomatic abutments in two patients were disconnected because of uncomfortable prostheses, although 84% of patients reported satisfaction levels of over 80%, and 31.8% awarded the maximum score for satisfaction (100%).

Bothur *et al.* (7) observed that during the evaluation period, all patients affirmed their dependence on speech at work, in their spare time, and in daily conversation. Speech is influenced by a number of factors, and difficulties can be initiated by alterations in the length, position, inclination and thickness of the teeth (7). Speech distortion increases rapidly when the palatal plate is made more than 1 mm thicker (8), and the palatal alterations in the present sample were obvious: satisfaction with speech was the lowest of all the parameters studied, although scores were nevertheless high (9.36 on the VAS). Lundqvist *et al.* (15) observed that following initial phonetic problems, 94% of the individuals considered themselves free from speech problems after three years of follow-up.

Further studies are needed to investigate the position of the implants at palatal level in relation to patient satisfaction with implant-supported fixed dental prostheses.

## Conclusions

No relationship was observed between prosthetic arm length and patient satisfaction.
